# Tumor Heterogeneity and the Immune Response in Non-Small Cell Lung Cancer: Emerging Insights and Implications for Immunotherapy

**DOI:** 10.3390/cancers17061027

**Published:** 2025-03-19

**Authors:** Michael S. Oh, Jensen Abascal, Austin K. Rennels, Ramin Salehi-Rad, Steven M. Dubinett, Bin Liu

**Affiliations:** 1 Department of Medicine, David Geffen School of Medicine at UCLA, Los Angeles, CA 90095, USA; michaeloh@mednet.ucla.edu (M.S.O.); jensenabascal@g.ucla.edu (J.A.); arennels@mednet.ucla.edu (A.K.R.); rsalehirad@mednet.ucla.edu (R.S.-R.); sdubinett@mednet.ucla.edu (S.M.D.); 2 Department of Medicine, VA Greater Los Angeles Healthcare System, Los Angeles, CA 90073, USA; 3 Jonsson Comprehensive Cancer Center, UCLA, Los Angeles, CA 90095, USA

**Keywords:** non-small cell lung cancer, heterogeneity, tumor evolution, immunotherapy

## Abstract

Resistance to immune checkpoint inhibitors (ICIs) represents a major challenge for the effective treatment of non-small cell lung cancer (NSCLC). Tumor heterogeneity has been identified as an important mechanism of treatment resistance in cancer and has been increasingly implicated in ICI resistance. The diversity and clonality of tumor neoantigens, which represent the target epitopes for tumor-specific immune cells, have been shown to impact the efficacy of immunotherapy. Advances in genomic techniques have further enhanced our understanding of clonal landscapes within NSCLC and their evolution in response to therapy. In this review, we examine the role of tumor heterogeneity during immune surveillance in NSCLC and highlight its spatial and temporal evolution as revealed by modern technologies. We explore additional sources of heterogeneity, including epigenetic and metabolic factors, that have come under greater scrutiny as potential mediators of the immune response. We finally discuss the implications of tumor heterogeneity on the efficacy of ICIs and highlight potential strategies for overcoming therapeutic resistance.

## Simple Summary

Immunotherapy has become a critical component of treatment for non-small cell lung cancer, but many tumors develop resistance to these therapies. In this review, we summarize recent studies showing that the presence of variability within the tumor, or intratumor heterogeneity, represents an importance source of resistance to immunotherapy. We discuss different types of heterogeneity—such as those arising from mutations, gene expression differences, and changes in metabolism—and explore how these traits interact with the immune system. Finally, we propose therapeutic strategies that could be used to combat the negative impact of high tumor heterogeneity.

## 1. Introduction

Cancer undergoes evolution throughout its development and progression in a process resembling natural selection. Somatic mutations accumulate in individual tumor cells, giving rise to distinct subclones that compete for dominance based on their relative fitness [1,2]. Clonal evolution begins early in tumorigenesis, with driver mutations often manifesting as homogeneous events prior to transformation to invasive disease [3,4]. Tumors then continue to experience complex genomic changes, including single nucleotide variants (SNVs), insertion–deletion events (indels), copy number alterations (CNAs), and whole genome duplications (WGDs) [2,5]. These alterations can either be clonal mutations observed throughout the tumor or subclonal mutations that are found in a subset of tumor cells. The variable accumulation of these changes leads to significant intratumor heterogeneity (ITH), which may encompass numerous spatiotemporal and biological parameters. The rate of mutation acquisition and the clonality of mutations varies among tumor types, with non-small cell lung cancer (NSCLC) typically possessing both a higher clonal and subclonal mutational burden than most other cancers [6]. NSCLC thus represents a disease in which ITH has the potential to play an outsized role in tumor progression and therapy response.

The immune system plays a pivotal role in shaping tumor evolution. Tumors can be recognized by the adaptive immune system due to the accumulation of tumor-specific mutations, which can potentially lead to the production of unique protein epitopes called neoantigens [7,8]. These neoantigens are displayed by major histocompatibility complex (MHC) molecules on antigen-presenting cells (APCs) to activate T cells, prompting an immune response against tumor cells that harbor those neoantigens. This immune surveillance acts as a selective pressure by pruning immunogenic subclones and fostering subclones capable of immune evasion, resulting in immune editing of the tumor. Immune escape mechanisms can arise early in tumor development and are often driven by characteristics inherent to the tumor [9,10], although primary and acquired mechanisms of immune resistance are complex and not yet fully characterized.

The emergence of immune checkpoint inhibitors (ICIs) in the treatment of NSCLC and other malignancies has highlighted the importance of the antitumor immune response. These therapies block immune regulatory signals, such as those mediated through programmed death (PD)-1 and cytotoxic T-lymphocyte-associated protein (CTLA)-4, which normally serve to restrain immune cytolytic activity against tumors [11]. ICIs have transformed the treatment landscape of NSCLC and are recommended for use as part of perioperative therapy in early-stage disease and in the first-line setting for metastatic disease [12,13]. Despite their success, ICIs are effective in only a minority of patients with NSCLC, and thus understanding resistance mechanisms is a major translational research goal in this field. Improved outcomes with ICIs have been linked to a high tumor mutation burden (TMB) and an increased number of neoantigens [14,15]. The mere presence of targetable neoantigens may be insufficient, however, if an immunosuppressive tumor microenvironment (TME) hinders their effective presentation to T cells by either APCs or tumor cells [16]. Moreover, T cells may be unable to fully eradicate tumors if their target neoantigens are not expressed by the majority of tumor cells. Only clonal neoantigens have been associated with favorable clinical outcomes following ICI therapy [17,18].

**Figure 1:**
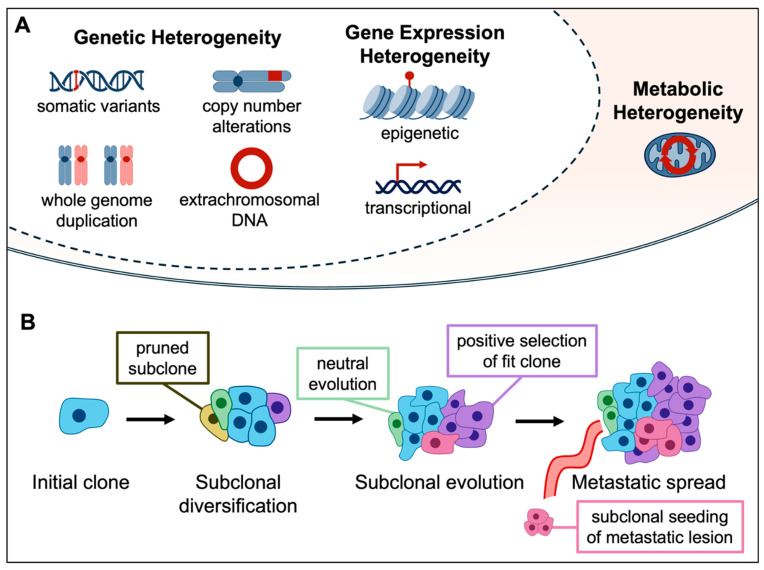
Sources of tumor-intrinsic ITH and effects on tumor evolution. (**A**) Different forms of tumor-intrinsic heterogeneity include genetic, gene expression, and metabolic factors. (**B**) Schematic of clonal evolution of the tumor over time. After tumor initiation, tumors experience branching evolution with the generation of new subclones. Fit subclones undergo positive selection and become dominant, while other clones undergo negative or neutral evolution. Other subclones can develop traits amenable to metastatic spread. ITH, intratumor heterogeneity.

The presence of ITH thus poses a potential barrier to effective immunotherapy. In this review, we delve into the factors contributing to ITH in NSCLC, examining how these elements influence the tumor-immune interface (Figure 1). We also discuss the impact of novel technologies on our ability to study ITH and explore possible therapeutic strategies for overcoming the clinical challenges created by ITH.

## 2. Genetic ITH and Clonal Evolution

The intrinsic complexity of tumors can lead to ITH spanning across multiple tumor attributes, including genetic alterations, gene expression profiles, and metabolic pathways. Of these factors, genetic features have been the most comprehensively characterized aspect of ITH. Tumor development is marked by constant mutagenesis, fueled by the inherent genetic instability of cancer cells. In NSCLC, this process begins in the early stages of tumor development, and heterogeneous cell populations have been found even in premalignant lesions [19]. Ongoing evolution during tumor progression leads to further subclonal variation driven by several mutagenic processes. Genetic diversity among subclones in NSCLC is shaped heavily by chromosomal events, such as CNAs [20,21]. Mutations can also accumulate due to established tumorigenic factors, such as tobacco exposure, while later-stage NSCLC tumors have been shown to exhibit a greater prevalence of SNVs generated by apolipoprotein B mRNA editing catalytic polypeptide-like (APOBEC) cytidine deaminase activity [20,22]. More recently, extrachromosomal DNA has been shown to impact cancer progression via oncogene amplification and immunomodulatory effects, representing yet another source of genetic ITH [23]. Inheritance of extrachromosomal DNA can occur both randomly and in a coordinated fashion, leading to additional complexities in tumor evolution [24].

The extent of subclonal neoantigen heterogeneity in NSCLC can be highly variable, with one study reporting that 10–78% of neoantigens are heterogeneously expressed within the tumor [18]. The prevalence of tumor subclones is determined based on selection driven by both intrinsic and extrinsic features. Mutations in oncogenic drivers or tumor suppressor genes can have a profound impact on clone fitness, though many other tumor mutations do not significantly affect subclonal selection. A large proportion of mutations have been shown to be neutral “passengers”, while mutations with minor functional consequences undergo only weak selection [25]. In fact, many subclones within heterogeneous tumors undergo neutral evolution [26], leading to the accumulation of subclones that are neither dominant nor immunologically pruned. Tumor evolution also entails complex processes not limited to simple competition among the fittest clones. Cooperation between two distinct subclones can occur, as reported in breast cancer [27,28] and small cell lung cancer [29], where subclones can secrete factors that enhance the propagation of other subclones. In melanoma, subclones with deleterious mutations in the interferon (IFN)-γ pathway are paradoxically well controlled by the immune system due to the downregulated tumor expression of programmed death ligand 1 (PD-L1), but these cells exhibit immune resistance when supplementary PD-L1 signaling is provided by nearby IFN-γ-intact subclones [30].

Phylogenetic reconstruction of subclonal evolution in both NSCLC and other tumor types generally reveals a branching evolutionary pattern, though alternative pathways, such as convergent evolution, have been observed [31,32]. The most extensive characterization of heterogeneity and clonal evolution in NSCLC has been conducted by the TRACERx consortium, which has compiled multi-region tumor sequencing in over 400 patients with early-stage disease [33]. Their analyses of resected tumors demonstrated that driver mutations tend to be early, truncal events that undergo clonal selection, as are mutations in key tumorigenic pathways, such as MYC [34]. Tumors then evolve further through an array of mutagenic changes, leading to subclonal expansions shaped by selective pressures [34]. Furthermore, a subset of subclones can acquire metastatic potential and seed new metastases [35,36], a paradigm supported by studies showing that metastatic lesions exhibit mutation profiles consistent with a subclonal origin from the primary tumor [37]. Though metastases frequently are homogeneous lesions originating from a single subclone, murine models have demonstrated the possibility of polyclonal seeding, such as by tumor clusters in breast cancer [38], and approximately 30% of metastases in NSCLC have been shown to be polyclonal in origin [36]. Heterogeneity thus extends beyond ITH to include differences between temporally and spatially distinct metastatic lesions.

## 3. Non-Genetic Sources of ITH

### 3.1. Gene Expression ITH

Tumor heterogeneity can encompass factors beyond the genetic profile of tumor subclones. In genetically homogeneous tumors with clonal driver mutations, diverse histologic types can be identified that are associated not with genetic but rather transcriptional and epigenetic changes [39,40]. Though chromosomal instability and copy number changes are major drivers of gene expression in tumors [41], phylogenetic analysis based on genetic factors is often unable to explain gene expression ITH [42]. Furthermore, genetic subclones do not consistently map onto gene expression patterns in NSCLC [43,44]. This discrepancy has been observed early in lung cancer evolution, and a variety of tumor-associated regulatory programs can even be seen in non-malignant tissue prior to cancerous transformation [43].

Unique transcriptional states can arise in small but highly impactful tumor subpopulations, underscoring the clinical importance of transcriptional ITH. During the progression of lung adenocarcinoma, the development of transcriptional ITH has been shown to be mediated by a high-plasticity cell state that promotes phenotypic diversity independently of genetic events [45]. Cell type-specific gene expression programs have been implicated in NSCLC tumorigenesis and malignant progression, including through the development of cancer stem cells (CSCs), which are multipotent, self-renewing tumor cells that have been linked to treatment resistance [46]. CSCs and other tumor cells can undergo transcriptional changes, such as epithelial-to-mesenchymal transition, to promote metastatic spread and further mediate resistance to therapies [47,48]. The ability to identify transcriptional subpopulations has become significantly easier due to the widespread adoption of single-cell RNA sequencing (scRNA-seq) techniques, which can characterize gene expression profiles of individual tumor cells. Sequencing data in a variety of oncologic contexts continues to be generated and can be expected to yield greater insights into interactions between gene expression and ITH.

Epigenetic regulation of gene expression has emerged as a critical contributor to ITH [49], including in lung cancer [50], and analyses that do not account for epigenetic factors can significantly underestimate transcriptional heterogeneity [44]. In Ewings sarcoma, the regulation of the disease-defining Ewing sarcoma breakpoint region 1 (EWS) Friend leukemia integration 1 (FLI1) translocation by DNA methylation has been shown to impact disease phenotype [51], demonstrating that even seemingly monolithic driver mutations are subject to epigenetic factors. Likewise, DNA methylation and histone acetylation exhibit significant heterogeneity across and within patients samples, as shown in studies of breast cancer [52] and pancreatic cancer [53]. Epigenetic ITH in NSCLC plays a pivotal role in tumor evolution, and the heterogeneity of DNA methylation has been associated with risk of progression in pre-invasive lesions [54]. The immune fitness of subclones is determined in part by epigenetic effects on neoantigen expression. The majority of clonal neoantigens in NSCLC are not uniformly expressed [55], and transcriptional variation of these neoantigens is often driven by methylation status as well as by subclonal mutations in epigenetic modifier genes [56]. Epigenetic phenotypes can also determine metastatic potential, and studies in murine lung cancer models have identified pro-metastatic regulatory programs that become dominant in metastatic lesions [57]. Due to their complexity and wide-ranging effects, epigenetic factors can exert significantly more influence on tumor phenotype than genetic variation [50], and thus further characterizations of epigenetic ITH are crucial to better understanding tumor evolution in NSCLC.

In addition to epigenetic processes, other mechanisms, such as RNA editing, also contribute to transcriptional diversity within tumors [56]. Tumor microenvironmental factors, such as heterogeneity in tumor hypoxia [58] and tumor endothelial cell phenotypes [59], can further affect gene expression. The importance of the TME is underscored by studies showing that metastases in the same organ share greater transcriptional commonality than more genetically similar metastatic tumors [60].

### 3.2. Metabolic ITH

Aberrant metabolism is a hallmark of cancer, with many tumors shifting towards reliance on glycolysis for energy production [61]. Studies have increasingly noted that patterns of metabolic adaption in tumors are highly heterogeneous. Early reports observed heterogeneity in intratumoral metabolism based on positron emission tomography (PET) scans, and greater ITH of fluorodeoxyglucose uptake on PET imaging has been associated with worse patient outcomes in melanoma [62], breast cancer [63], and NSCLC [64].

Novel techniques for directly measuring metabolic activity have facilitated additional studies on metabolic ITH. The use of a glucose fluorescence resonance energy transfer (FRET) biosensor in breast cancer models showed that glucose concentration was heritable but diverged over time, even among genetically identical cells derived from a single clone [65]. Voltage-sensitive PET tracers have been utilized to characterize the heterogeneity of mitochondrial function and metabolic dependencies in live murine lung cancer tumors [66,67]. Direct assessments of metabolic activity in patient samples have been limited due to challenges in studying metabolism without living tissue, but creative strategies in the perioperative setting have provided valuable observations. For instance, 13C-labeled glucose infusion prior to lung tumor resection revealed heterogeneous dependence on either glycolysis or oxidative phosphorylation, and these metabolic preferences correlated with findings on dynamic contrast-enhanced MRI [68]. Technical advances in metabolic imaging, in conjunction with more traditional bioenergetic profiling methods, may improve our understanding of the mechanisms of metabolic ITH.

Differences in metabolic parameters have been linked to diverse tumor behaviors, which have important implications for tumor evolution. In patient-derived xenografts of melanoma, tumor cells with a higher expression of monocarboxylate transporter 1 (MCT1), and thus a metabolic profile skewed by increased lactate uptake, exhibited greater metastatic potential [69]. Significant metabolic heterogeneity has been reported in NSCLC, including tumor-specific features that signify potentially targetable metabolic vulnerabilities. Squamous cell carcinoma tumors have been shown to be more critically reliant on glycolysis than adenocarcinoma tumors, and these histologic subtypes accordingly display different responses to glycolytic inhibition [70]. Greater expression and dependence on sodium-dependent glucose transporter 2 (SGLT2) has been observed in early-stage NSCLC lesions as compared to late-stage disease, indicating a temporal component of metabolic heterogeneity [71]. Intratumoral variation in these metabolic attributes has been demonstrated in NSCLC [68], while a more comprehensive characterization of metabolic ITH remains open for future exploration.

Some metabolic characteristics in NSCLC have been linked to driver mutations [72], but much of metabolic ITH extends beyond genetic causes. Tumor metabolism can notably be influenced by vascular and hypoxic changes in the TME. Phenotypically different subclones in melanoma xenografts exhibit varying levels of dependence on angiogenesis, often based on exposure to hypoxic conditions [73]. Differences in local hypoxia and vascular integrity have been predicted to drive a significant amount of metabolic ITH in computational models [74]. Crosstalk between different forms of heterogeneity further shapes tumor evolution, as metabolic byproducts can alter DNA methylation and histone acetylation to impact epigenetic heterogeneity [53,75]. It has thus become clear that ITH involves numerous complex and interwoven factors that affect tumor progression and treatment response, and efforts to better understand these interactions may provide important insights into oncologic therapies.

## 4. ITH and the Immune Microenvironment

**Figure 2:**
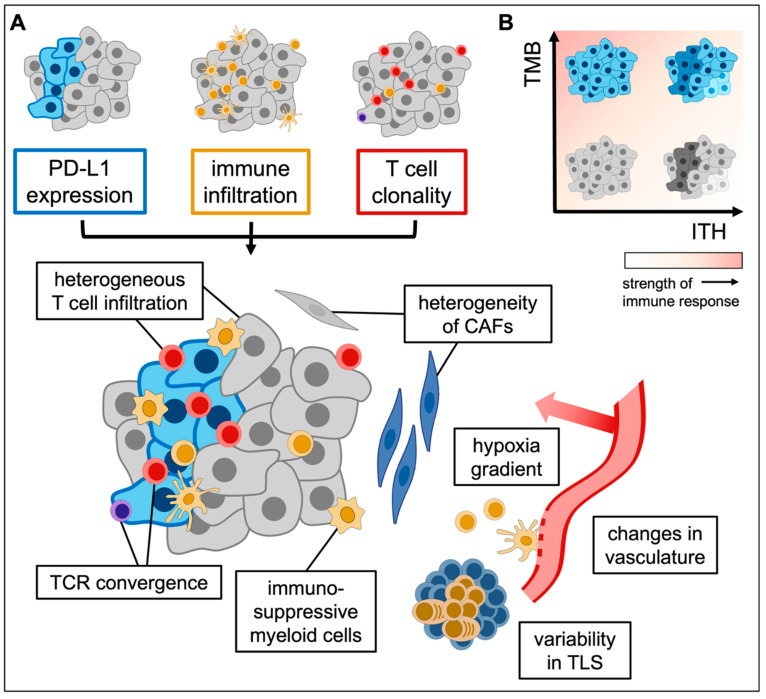
Immune ITH. (**A**) Immune-related ITH can encompass multiple aspects, including immune cell infiltration, PD-L1 expression, and T cell clonality, which interact and overlay on tumors to form a heterogeneous tumor immune microenvironment. Immune ITH can be impacted by tumor-intrinsic and -extrinsic factors, the latter including stromal and vascular changes. (**B**) Immune response to tumors can be stratified based on two related yet independent variables: TMB and ITH. Tumors with high clonal neoantigen burden, indicating a high TMB and low ITH state, are most likely to respond to immunotherapy. ITH, intratumor heterogeneity; PD-L1, programmed death ligand 1; TMB, tumor mutational burden; TCR, T cell receptor; CAF, cancer-associated fibroblast; TLS, tertiary lymphoid structure.

Throughout tumorigenesis, cancer cells experience dynamic cycles of immune surveillance and escape, which shape both the tumor and the immune microenvironment [76]. The level of immune cell infiltration and activity can vary within the tumor, representing immune-related ITH that co-exists with tumor-intrinsic heterogeneity (Figure 2A). The immune response is often affected heavily by more uniform host-dependent factors, leading to low immune ITH in several cancer types, such as von Hippel Lindau disease (vHL)-related renal cell carcinoma [77] and intrahepatic cholangiocarcinoma [78]. In contrast, studies have demonstrated that many immune characteristics are highly heterogeneous in NSCLC.

### 4.1. Heterogeneity of Immune Biomarkers in NSCLC

Specific interest has been directed towards quantifying the presence of tumor-infiltrating lymphocytes (TILs) and tumor expression of PD-L1, both of which are immune biomarkers associated with response to ICIs [15,79,80]. Histologic studies of NSCLC tumors have shown that there is significant heterogeneity in TIL distribution [81], and thus the method and location of biopsy sampling can affect both TIL assessments and their clinical relevance. For example, T cell infiltration evaluated in biopsies taken from the tumor center may serve as a better predictor of patient survival than infiltration in samples from other regions [82]. Numerous studies have also highlighted inconsistencies in PD-L1 testing, which sometimes occur due to inter-assay differences but more often due to intrinsic heterogeneity of the tumor [83]. Immune heterogeneity exists both within and among tumors, and concordance of PD-L1 expression between primary tumors and metastatic lymph nodes is less than 50% [84,85]. PD-L1 expression on lymph node metastases is in fact less predictive of response to ICIs than expression levels in the primary lung tumor or distant metastatic sites [86,87]. The heterogeneity of these parameters can also correlate with existing categorizations of NSCLC, such as histological subtypes. Adenocarcinomas have been shown to have greater ITH in regard to PD-L1 positivity and immune cell phenotypes [88,89], despite being more likely to possess clonal driver mutations. These findings together demonstrate that the antitumor response in NSCLC is highly heterogeneous and can even affect the reliability of immune biomarker testing.

### 4.2. Determinants of Immune-Related ITH

The advent of technologies, such as scRNA-seq and T cell receptor sequencing (TCR-seq), has provided further insights into immune-related ITH. T cell clones are often confined to specific regions of a tumor, with one study in lung adenocarcinoma showing that fewer than 14% of T cell clones were found in all parts of the tumor [90]. Expanded TCRs in the same tumor region have been shown to converge towards similar sequences [91] or similar binding affinities [92], suggesting that distinct T cell clones in a tumor region tend to target a common set of neoantigens. T cell clonality has consistently been shown to mirror the clonality of immunogenic tumor neoantigens, underscoring the reciprocal adaptations between evolving tumors and the immune system [93]. Neoantigen profiles specific to particular regions of NSCLC tumors are accompanied by a unique TCR repertoire [94]. Conversely, increased T cell infiltration has been associated with lower genetic ITH, likely reflecting robust immunoediting by the antitumor immune response [95].

The immunologic impact of neoantigens also depends on the effectiveness of their presentation to immune cells. Mutations that are poorly presented by the host MHC are found more frequently in tumors, indicating that immunoediting heavily relies on intact mechanisms of antigen presentation [96]. Certain human leukocyte antigen (HLA) types preferentially display highly immunogenic neoantigen peptides, leading to improved outcomes with ICI therapy [97]. Loss of heterozygosity of HLA alleles accordingly represents another important source of ITH and an immune escape mechanism in NSCLC tumors [98]. The immunogenicity of neoantigens is, therefore, determined by both their intrinsic properties and MHC-mediated presentation. In addition, it has become evident that the co-expression patterns of neoantigens can affect the resulting immune response. In murine models of lung adenocarcinoma, clonal co-expression of strong and weak neoantigens synergistically improved the T cell response, while the subclonal expression of an immunodominant neoantigen led to immune evasion via the outgrowth of subclones lacking this neoantigen [99]. The importance of neoantigen co-expression was further demonstrated in lymphoma models, where T cell response against immunogenic fluorescent proteins occurred only in the presence of other strong antigens [100].

Though the mosaicism of neoantigen presentation plays a critical role in determining the immune response, many other tumor characteristics are also correlated with immune heterogeneity. Increased transcriptional ITH is associated with immune cell heterogeneity at all stages of tumor development [101,102]. In scRNA-seq analyses, transcriptional ITH has specifically been shown to positively correlate with neutrophil and macrophage frequency and negatively correlate with plasma cell frequency [103,104]. The activation of certain tumor pathways can affect immune infiltration, such as in ovarian cancer, where heterogeneous Wnt signaling promotes local immune exclusion [105]. Stem-cell-like tumor signatures across multiple cancers have been associated with both greater ITH and immunosuppressive phenotypes [106]. The relationship between tumor transcriptional profiles and antitumor immunity is becoming increasingly apparent with these studies.

### 4.3. Spatial Components of Immune-Related ITH

The spatial architecture of the TME is of particular importance when discussing heterogeneity within the immune compartment. For example, tertiary lymphoid structures (TLSs) are organized immune cell aggregates that have been associated with antitumor immunity and favorable clinical outcomes in NSCLC and other cancers [107], but they can be highly heterogeneous in terms of frequency, location, and function [108]. The spatial distribution of T cells within the TME can also denote clinically significant information [109] and is determined in part by non-genomic factors, such as interactions with functionally active myeloid cells [110]. Spatial transcriptomic analyses have revealed that tumor cells express immune-related genes along spatial gradients that correlate with immune cell density [111]. In addition, immune cell ITH is influenced by the spatial relationship between tumors and their accompanying stromal elements, including the tumor vasculature and cancer-associated fibroblasts (CAFs). In breast cancer, CAFs from different regions possess distinct phenotypes and were shown to differentially affect immunologic characteristics and clinical outcomes [112].

The tumor-immune landscape varies across organs and metastatic sites, and tissue-specific factors are key to understanding these disparities [113]. The blood–brain barrier is well known to modulate the immune environment within the brain, and brain metastases exhibit lower T cell infiltration than paired primary lung tumors [114]. A study of synchronous metastases in melanoma showed very low (<8%) overlap in T cell clones [115], further underscoring the marked immune ITH that exists between metastatic lesions. The immune milieu of the lung, which is subject to a variety of inflammatory insults and involves unique players such as alveolar macrophages, can also exert a distinct influence on tumorigenesis for NSCLC [116]. These findings suggest that interactions with tumor extrinsic factors play important roles in determining immune-related ITH.

## 5. Clinical Impact of ITH

The clinical importance of ITH has been well established in certain contexts, such as therapeutic targeting of driver mutations. Highly heterogeneous tumors possess a diversity of genetic alterations, which can include oncogenic variants that mediate resistance to targeted therapies [117], such as in EGFR-mutated NSCLC [118] or BRAF-mutated melanoma [119]. The link between ITH and antitumor immunity remains less clear, given the ability of the immune system to theoretically adapt to changes in evolving tumors. Evidence in NSCLC and other cancers nevertheless has suggested that ITH represents a barrier to effective antitumor immunity. Mechanistically, high ITH may make it difficult for the immune system to adequately target each subclone. Preclinical models have suggested that there is a minimum clonal fraction required for proper immune rejection of a subclone, demonstrated by highly immunogenic neoantigens evading immune targeting if present at a low enough proportion [120]. Excessive heterogeneity, on the other hand, may be detrimental to tumor fitness. A pan-cancer analysis showed that ITH was associated with worse patient survival only up to a point, and the prognosis was worst in the patients with tumors harboring an intermediate level of CNAs [121]. These results imply that there is an optimal level of ITH for tumors to most effectively evade the immune response.

In terms of clinical impact, high ITH has been associated with reduced T cell infiltration and worse patient outcomes in multiple tumor types, including breast cancer [122], ovarian cancer [123], hepatocellular carcinoma [101,124], and melanoma [115,125]. In a study of resected lung adenocarcinomas, greater genetic ITH was associated with an increased risk of relapse [126]. Certain forms of genetic heterogeneity appear to better correlate with prognosis, possibly because some alterations are evolutionarily neutral. Elevated copy-number heterogeneity but not mutational ITH in NSCLC was associated with an increased risk of recurrence or death in the TRACERx cohort [36,127]. Subclonal WGDs and recent large subclonal expansions were also associated with poorer prognosis, with the latter being the strongest predictor of relapse in a multivariate analysis [36]. In addition to the link between genetic ITH and worse clinical outcomes, DNA methylation-based ITH has been associated with poor prognosis [128]. The spatial distribution of tumor clones may also be important, as illustrated by a study showing that random rather than clustered distribution of tumor clones was associated with worse clinical outcomes [129]. These data support the notion that increasing the distance between viable T cell targets introduces greater opportunity for physical or soluble immune obstructions.

ITH has been shown to not only affect antitumor immunity but also correlate with response to ICIs. In a meta-analysis of sequencing data from multiple tumor types after ICI therapy, clonal but not subclonal TMB was identified as the leading predictor of response [17]. This result was independent of any association between total TMB and ICI response. TMB has previously been established as a biomarker of response for ICIs, and both pembrolizumab and nivolumab have been approved for the treatment of tumors with high TMB [130,131]. ITH and TMB both derive from genomic instability and are often linked, but these two variables do not strongly correlate with each other [132,133] (Figure 2B). High TMB indicates a surfeit of neoantigens that can be targeted by the immune system but does not capture the clonality of those neoantigens. Response to ICIs in NSCLC specifically has been associated with clonal neoantigen burden [18]. ITH thus represents a predictive factor that can impact immunotherapy beyond TMB, and prognosis within low-TMB tumors can be stratified by the level of ITH [132]. Meanwhile, the successful use of ICI can impact tumor clonal composition. For example, a favorable response to nivolumab in melanoma was associated with mutational contraction and loss of clones [134].

Immune ITH similarly has demonstrated correlations with clinical outcomes, though these connections are less robust than with tumor-intrinsic ITH. Studies have yielded conflicting data on the relationship between TCR clonality and clinical prognosis [90,91]. Comprehensive immune analyses may be more informative than a singular focus on the T cell response, and a study on RNA sequencing in lung cancer demonstrated an association between total immune cell diversity and improved clinical outcomes [135]. Ascertaining spatial patterns within tumors will also be critical to understanding the immune response to ITH. Multiplex immunofluorescence of NSCLC tumors has shown that a higher density of CD8+ cytotoxic T cells in stromal tissue surrounding tumor nests was associated with improved survival after ICIs [136]. Certain immune niches, such as those defined by specific T cell–myeloid interactions using single-cell spatial transcriptomics, have been associated with clinical response [110,137]. An improved understanding of all aspects of immune ITH may thus be crucial to identifying which factors affect patient survival and response to immunotherapies.

## 6. Current Techniques for Understanding ITH

### 6.1. Studying ITH with Improved Models

Preclinical studies of heterogeneity remain limited by the paucity of proper models to interrogate ITH. Murine models may often fail to recapitulate the heterogeneity of patient tumors, in part due to lower genomic instability in models that are established using genetically engineered oncogenic drivers [138]. Carcinogens such as chemical mutagens or UV radiation may be utilized to introduce greater variability in these models [139,140], as can directed mutagenesis of DNA polymerase or DNA repair mechanisms [141]. Though these methods clearly increase the TMB, it is not clear that the resulting tumors have higher subclonal heterogeneity. Patient-derived xenografts have been shown to better reflect the molecular heterogeneity of these tumors and may be more suited for certain studies [142]. Organoids established from patient tumors similarly can retain the genomic ITH of parental tumors and have been used to screen potential treatments [143].

Several groups have successfully modulated ITH in murine models by isolating single-cell clones and recombining them to form tumors of varying heterogeneity. Combinations of single-cell clones from a melanoma cell line were used to demonstrate that tumors containing a greater number of clones or more genetically different clones were more aggressive [139]. A similar model in pancreatic cancer was used to show that tumors established from subclones exhibited different immune infiltration states, even though they derived from a common parental cell line [144]. Mixing these subclones into heterogeneous tumors revealed that T cell low subclones acted dominantly to exert an immunosuppressive phenotype. Contrasting subclones can alternatively be generated by introducing rationally chosen antigens—such as those with varying immunogenicities—into a tumor cell line, followed by combining the resulting subclones into heterogeneous tumors [99,120].

Other models have been designed to generate intrinsic heterogeneity in vivo rather than manipulating individual clones. For example, an autochtonous NSCLC model has been reported that uses CRISPR/Cas9 to generate indels and trace resulting subclones [145]. A multi-recombinase strategy has been utilized to induce sequential mutations and mimic subclonal oncogenesis in leukemia [146]. Subclones can also be engineered to exhibit specific phenotypes, such as in a breast cancer xenograft model where subclones were modified to secrete factors implicated in tumor progression. In this model, an IL-11-expressing subclone was found to bolster tumor growth and foster clonal diversity despite remaining a numerically minor clone, highlighting an example of clonal cooperation [147]. Comparable models in NSCLC may reveal interactions among tumor subclones that can shed light on mechanisms of ITH-driven tumor progression.

Cell labeling is an essential component of models in ITH studies due to the need to trace subclones and determine subclonal evolution in response to treatments. A variety of methods exist to fluorescently label tumor cells, including traditional methods of transduction with viral vectors as well as genetic engineering of murine models to label tumors in vivo [148]. For example, the Confetti model utilizes a Cre reporter system to induce the expression of a randomly selected fluorescent protein in tumor cell lines [149]. This model has been utilized to illustrate the polyclonal characteristics of de novo colorectal tumors [150] and pancreatic cancer metastases [151], as well as to study the evolution of putative CSCs in mouse intestinal adenomas [152]. Tracking of subclones can also be performed through DNA barcoding, including in the context of scRNA-seq [119,153]. Sequencing-based methods may avoid issues caused by the potential immunogenicity of fluorescent proteins [154] and allow simultaneous characterization of tumor-reactive T cells and other immune components [155], though they may make it difficult to ascertain spatial information regarding subclones. Ultimately, models that combine accurate labeling techniques with the ability to modulate tumor ITH are lacking in NSCLC, and the development of such models is needed to facilitate future studies of ITH.

### 6.2. Assessing ITH in Clinical Samples

Translating an improved understanding of ITH to clinical application will require the ability of clinicians and scientists to assess ITH in patients. The most accurate method for assessing ITH and tracking subclones is via analysis of whole tumors, such as from surgical resections. Biopsies can yield representative information but are susceptible to sampling error. Nevertheless, advances in sequencing and multiomics have drastically increased the ability to assess tumor evolution and immune responses. Next-generation sequencing (NGS) methods have improved to the point of being utilized clinically to make treatment decisions in NSCLC [156]. In the research setting, the lowered cost of NGS has facilitated the generation of large datasets that can be leveraged to study ITH [157]. Progress in spatial transcriptomics techniques has accelerated [158], and these methods can now be used to infer single cell-level gene expression [159] and develop 3D reconstructions of the tumor transcriptome [111]. Automated imaging processing has proven to be useful in analyzing spatial data, such as in an analysis of histologic images of breast cancer that showed a correlation between high cellular heterogeneity and poor prognosis [160]. Applications of artificial intelligence (AI) remain in their infancy but have shown great promise in enhancing image processing [158,161]. For example, an AI-based identification pipeline of cell types from hematoxylin and eosin (H&E) images was able to identify immunologically cold and hot regions in NSCLC tumors, which correlated with clonal diversity and clinical outcomes [162].

Circulating tumor DNA (ctDNA) offers a less invasive method to profile tumor mutations and has the potential to reveal mutations found in multiple tumor locations, rather than being confined to a single biopsy site. Analysis of ctDNA involves sequencing DNA fragments released by tumors into circulating plasma [163,164]. CtDNA analyses have facilitated the identification of resistance mechanisms to EGFR tyrosine kinase inhibitors [165]. They detect minimal residual disease after surgical resection [166] and clarify response to ICIs [167]. In patients whose baseline mutations in the primary tumor are known, ctDNA studies can identify metastatic subclones and reconstruct clonal phylogenetic evolution [166]. However, there are limitations to the sensitivity of ctDNA, and several studies have demonstrated discordance between ctDNA and tumor sampling in regard to evaluating TMB and ITH [168,169]. Caution must thus be employed when interpreting assessments of ITH using current technologies. Future strategies for incorporating ITH in clinical applications will need to take into account the limitations of the measurement method.

## 7. Therapeutic Strategies to Overcome ITH

**Figure 3:**
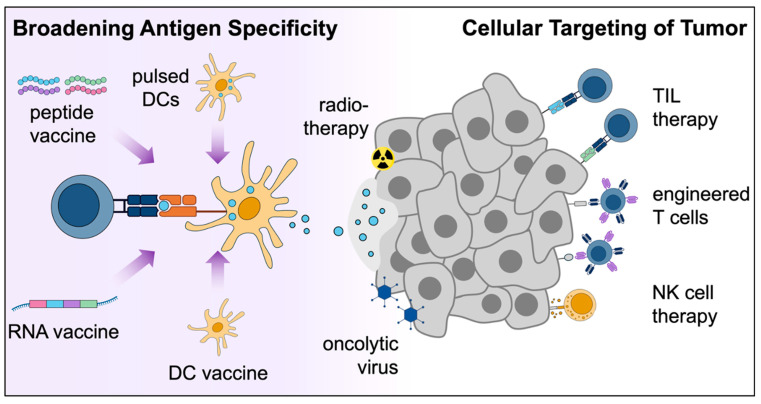
Potential therapies to overcome ITH. Overview of therapeutic strategies to overcome ITH-related tumor resistance. These treatments can generally be categorized as broadening the T cell response against more tumor antigens or maximizing immune cell cytolytic functions against a variety of tumor subclones. ITH, intratumor heterogeneity; DC, dendritic cell; TIL, tumor-infiltrating lymphocyte; NK, natural killer.

Several strategies have emerged to overcome ITH in the setting of targeted therapies, such as the use of combinations of inhibitors to preempt resistance. These strategies are less applicable to immunotherapies due to the complexities of immune escape mechanisms. However, in a concept analogous to using combination therapies against multiple molecular targets, immunotherapies can be designed to expand the TCR repertoire against more neoantigens. Various treatment modalities have been proposed to achieve this goal, including neoantigen-focused vaccination strategies and cellular therapies (Figure 3). The former attempts to broaden the scope of targeted neoantigens and promote epitope spreading while the latter seeks to harness the full capabilities of an immune cell to overcome immunotherapy resistance.

### 7.1. Vaccination Strategies

Vaccines composed of tumor neoantigen peptides have been investigated in murine models and have demonstrated the ability to elicit durable neoantigen-specific T cell responses [170,171,172]. A phase Ib clinical trial of a neoantigen peptide vaccine in combination with first-line chemo-immunotherapy for advanced NSCLC demonstrated the clinical feasibility of this strategy, and correlative studies provided evidence for the induction of both neoantigen-specific T cell responses and epitope spreading [173,174]. RNA vaccines encoding neoantigens can be used instead of injecting peptides and have the advantage of lower cost and ease of delivery. The efficacy of an RNA-based vaccine was demonstrated in melanoma, in which lipid nanoparticles with mRNA encoding up to 34 neoantigens were administered to patients with high-risk disease after complete resection. The combination of the vaccine plus pembrolizumab significantly improved recurrence-free survival compared to pembrolizumab alone [175]. Proof of concept for RNA vaccines in NSCLC has been demonstrated in murine models, and clinical trials are currently in progress [176].

Despite the optimism surrounding these therapies, limitations exist due to the need to obtain sufficient tissue and identify neoantigens. In contexts where surgical resection is not indicated, such as metastatic disease, biopsies may not adequately represent the full neoantigen spectrum. These methods also rely on the successful prediction of neoantigens from somatic variants, which has improved in recent years but remains a computational inference. Furthermore, studies in murine models have suggested that strategically targeting specific neoantigens, such as subdominant clonal mutations, may be needed to maximize the impact of neoantigen-based therapies [99]. Directing treatment against a dominant clonal neoantigen can additionally lead to tumor resistance via neoantigen loss, as illustrated in a case of EGFR-mutated NSCLC that lost the EGFR driver mutation in response to an EGFR neopeptide vaccine [177]. There is thus significant time and cost involved in manufacturing these personalized therapies, and the optimal strategy for neoantigen selection remains uncertain, especially when ITH is taken into consideration.

The presentation of neoantigen peptides to T cells in tumors is performed predominantly by dendritic cells (DCs) [178], leading to considerable interest in utilizing DCs as a form of immunotherapy, including for NSCLC [179]. DCs can be pulsed with tumor lysate or neoantigen peptides, which have been shown to promote long-lived neoantigen-specific T cell responses [92,180]. The first DC vaccine approved for clinical use was sipuleucel-T, which was developed for prostate cancer as an autologous DC product that is loaded with the antigen prostatic acid phosphatase and activated with the cytokine GM-CSF. Sipuleucel-T was shown to confer improved overall survival (OS) compared to placebo with a hazard ratio of 0.78, indicating a 22% reduction in the risk of death [181]. Various strategies for DC vaccination have been attempted in NSCLC, but none have displayed sufficient efficacy to warrant clinical application. Our group has investigated non-pulsed DCs as intratumoral vaccines that can take advantage of native tumor antigens and boost antigen presentation, leading to the induction of both local and systemic tumor-specific T cell responses [182]. We have also genetically modified DCs to secrete cytokines and other pro-inflammatory factors, thereby augmenting the immune response beyond antigen presentation [183,184,185]. In addition to in situ vaccination with DCs, alternative approaches can be used to promote DC functions. Oncolytic viruses can induce tumor cell lysis and promote antigen presentation of released neoantigens. Talimogene laherparepvec (T-VEC) is an intratumorally delivered oncolytic virus that expresses GM-CSF to promote APC functions and has been approved for use in advanced melanoma [186,187]. T-VEC exhibited improved efficacy compared to GM-CSF injection alone in regard to both the objective response rate (ORR, 16.3% vs. 2.1%) and median OS (23.2 vs. 18.9 months) [187]. No viral therapies have been approved for NSCLC, but their potential efficacy has been demonstrated in preclinical studies [188,189].

### 7.2. Cytolytic Cellular Therapies

Many vaccine therapies rely on neoantigen dependence of the immune response and thus may still be susceptible to resistance mediated by genetic ITH. Cytolytic cellular therapies offer more direct tumor-killing mechanisms that can circumvent issues caused by high tumor heterogeneity. TILs from patient tumors can be expanded and re-infused, representing a novel immunotherapeutic strategy that can potentially ameliorate certain aspects of immune-related ITH, such as the spatial heterogeneity seen among T cell clones. The TIL therapy lifileucel has been granted accelerated approval based on phase 2 trial results demonstrating an ORR of 31.5% in patients who had previously progressed on anti-PD-1 therapy [190]. This same TIL platform has also yielded an ORR of 21.4% in ICI-refractory NSCLC, including responses in patients with PD-L1 negative and/or low TMB tumors [191]. Despite these early promising results, it is not clear if the T cell population derived from a limited region of the tumor would include a sufficiently broad TCR repertoire to overcome high neoantigen ITH. The composition of the TCR repertoire of TILs also changes during T cell expansion, often shifting away from dominant T cell clones that may be more relevant to tumor control [192].

Chimeric antigen receptor (CAR) T cells are T cells engineered against a tumor-associated antigen and have become widely used for hematologic malignancies [193]. By focusing on a commonly expressed cell surface antigen rather than a genomic alteration, CAR-T cells avoid issues related to neoantigen ITH. The ability of CAR-T cell therapy to be effective against solid tumors has been demonstrated by afamitresgene autoleucel. This product targets the MAGE-A4 antigen and achieves an ORR of 37% in heavily pre-treated patients who have qualifying HLA-A*02 alleles [194]. As with all approved CAR-T cell products, there was a high proportion of patients experiencing inflammatory toxicities, such as cytokine release syndrome, but most instances were low-grade events. No such therapies have been approved for NSCLC, but early-phase trials of CAR-T cells targeting EGFR [195] and ROR1 [196] have been conducted. Importantly, solid tumor targets often display differential expressions in target–antigen density across the whole tumor. Cancers can specifically evade CAR-T cell therapy through antigen loss [197], and thus these treatments are not fully exempt from ITH-based resistance. To address this challenge, affinity tuning of CAR molecules has been explored as a strategy to enhance CAR-T cell responses against tumor cells with low antigen density [198,199]. The use of a combination of targets may also mitigate these concerns, and dual-targeting CAR-T cells are in development for several cancer types [200,201]. In a preclinical study, T cells engineered with multiple bispecific antibodies showed promising efficacy against patient-derived NSCLC xenografts [202], further supporting a potential role for multifactorial cellular therapies.

Alternatively, innate immune cells, such as natural killer (NK) cells, may be able to counteract immunologic resistance due to their ability to target tumors independently of neoantigens. NK cells specifically have the capacity to kill tumor cells that have lost or downregulated MHC expression, which is a well-described immune evasion mechanism [203]. The feasibility of NK-based therapies has been demonstrated using both expanded NK cells [204] or NK cells engineered against a tumor-specific target [205]. Despite these promising advances, cellular therapies for NSCLC remain in the early stages, and the potential role of these treatments in overcoming ITH-related resistance is an underexplored question.

Finally, the inherent nature of ITH suggests a role for combination therapies in order to target multiple subclones and prevent the outgrowth of resistant phenotypes. Broadly active treatments, such as radiotherapy or tumor debulking surgery, may theoretically reduce ITH and ameliorate heterogeneity-mediated resistance [206]. Cytoreductive surgery has improved the efficacy of ICIs in murine models of NSCLC [207], though clinical data in this regard is lacking. The aforementioned therapies can also be utilized in combination. Promising efficacy has been observed in strategies such as the joint administration of a DC vaccine and an oncolytic virus [208] or the combination of CAR-T cell therapy with cancer vaccines [209,210]. The induction of epitope spreading has been reported using a pharmacologic stimulator of interferon gene (STING) agonists with CAR-T cells [211]. Additionally, NK cells are capable of CD16-mediated antibody-dependent cellular cytotoxicity and may synergize with tumor-specific targeting via monoclonal antibody therapy [212]. Attacking tumors utilizing multiple aspects of the antitumor immune response may, therefore, be able to circumvent individual immune evasion mechanisms and minimize the detrimental impact of ITH.

## 8. Conclusions

Significant preclinical and clinical evidence has accumulated in support of ITH representing a tumor resistance mechanism against immunotherapies. The specific mechanisms that underpin ITH-mediated immune escape remain insufficiently characterized. The widespread use of single-cell sequencing techniques and spatial multiomics approaches can be expected to generate a wealth of data that can further elucidate the effects of various forms of ITH. Underexplored areas, such as metabolic ITH, warrant more detailed exploration. There is also a clear need to develop more models that control for ITH to better assess the interaction between ITH and novel therapies. Progress in these fields should facilitate an improved understanding of ITH that moves past the identification of subclones and instead characterizes clonal dynamics and immune–clone interactions, such as subclone cooperation or neoantigen dominance. A critical potential advance in this field would be the identification of a predictive rather than a prognostic biomarker of ITH that can more accurately guide treatment decisions.

ITH should thus be considered in designing immunotherapies and choosing directions for future research. There have been exciting advances made in the realm of neoantigen-targeting therapies, including peptide and mRNA vaccines, as well as cellular therapies. However, efforts to show how these therapies interact with ITH have been limited, restraining current understanding of how best to employ these treatments. Ideally, mechanistic studies of these therapies would include ITH analyses and explore which aspects of heterogeneity mediate resistance or response. The tremendous progress in immunotherapies offers a unique opportunity to understand this important aspect of tumor biology and guide future directions to improve treatments for NSCLC.

## Data Availability

No new data were created or analyzed in this study. Data sharing is not applicable to this article.
